# Comparison of Emergency Department Patients with Salpingitis and Oophoritis with and without Documented Social Determinants of Health

**DOI:** 10.5811/westjem.48931

**Published:** 2026-03-01

**Authors:** Cassandra Farber, Priya Devanarayan, Gavin Schaefer-Hood, Hayes Stancliff, Catherine Marco

**Affiliations:** * Penn State College of Medicine, Hershey, Pennsylvania; † Penn State Health Milton S. Hershey Medical Center, Department of Emergency Medicine, Hershey, Pennsylvania

## Abstract

**Introduction:**

Social determinants of health (SDoH) have emerged as a critical focus of research due to their significant impact on clinical outcomes; however, there is a gap in research specific to women’s health. Understanding the factors underlying trends in gynecologic emergency diagnoses requires a more comprehensive examination of SDoH. In this study we characterize the demographic and clinical profile of patients with documented SDoH *International Classification of Diseases, 10^th^ revision* (*ICD-10*), Z codes (Z55-Z65) who presented to the emergency department (ED) with salpingitis and oophoritis, and explore patterns of healthcare utilization and management.

**Methods:**

In this retrospective cohort study we used TriNetX Research Network data to compare adult females (18–49 years of age) presenting to the ED with diagnosed salpingitis and oophoritis between January 1, 2000–January 1, 2024, by presence or absence of SDoH Z codes. Propensity score matching balanced baseline demographics and comorbidities. The outcomes assessed one year from ED presentation included surgical intervention, hospital admission, ED revisits, utilization of critical care service, analgesic use, and new mental health diagnoses such as anxiety, post-traumatic stress disorder, and depression. Risk analyses compared outcome proportions between cohorts, reported as risk ratios (RR) with 95% confidence intervals.

**Results:**

Before propensity score matching, the proportion of the initial cohort that had at least one SDoH Z code was 11.9%. Following propensity score matching, we analyzed 5,570 patients, 50% of whom had documented SDoH Z codes. We found that 10.2% of patients with documented SDoH Z codes received surgery compared to 15.0% of patients without (RR, 0.679; 95% CI, 0.577–0.799, P < .001). On the contrary, 45.7% of patients with Z codes were hospitalized compared to 34.3% without (RR, 1.333; 95% CI, 1.248–1.423, P < .001). Of patients with SDoH Z codes, 58.1% revisited the ED compared to 45.2% without (RR, 1.287; 95% CI, 1.222–1.355, P < .001). 4.4% of patients with Z codes required critical care services compared to 2.5% without (RR, 1.757; 95% CI, 1.317–2.345, P < .001). Lastly, patients with SDoH Z codes experienced new mental health diagnoses. This included 8.4% with Z codes diagnosed with depression (RR, 1.890; 95% CI, 1.432–2.495, P < .001) compared to 4.6% without, 11.1% with Z codes diagnosed with anxiety (RR, 1.565; 95% CI, 1.241–1.973, P < .001) compared to 7.1% without, and 2.7% with Z codes diagnosed with post-traumatic stress disorder (RR, 3.026; 95% CI, 1.897–4.826, P < .001) compared to 0.9% in patients without documented Z codes.

**Conclusion:**

Patients with documented *ICD-10* Z codes for social determinants of health were less likely to receive surgery but were associated with increased ED repeat visits, hospitalization, need for critical care, and mental health conditions. These findings highlight the clinical relevance of SDoH in acute care utilization and patient outcomes, underscoring the importance of routine screening and documentation of SDoH in electronic health records. Addressing underlying social needs may be a key strategy in reducing healthcare burden and improving long-term outcomes for vulnerable populations.

## INTRODUCTION

Social determinants of health (SDoH) have emerged as a critical focus of research due to their significant impact on clinical outcomes. The World Health Organization (WHO) defines SDoH as the conditions in which an individual’s access to money, power, and resources influences health equity.^1^ These conditions include place of residence, work, age, sex, race, and ethnicity.^1^ In general, lower socioeconomic status is associated with worse health outcomes such as a higher risk of illness and death.^1^ When it comes to disparities related to sex, there remains a gap in research specific to women’s health, including research dedicated to conditions that disproportionately impact women.^2^ Emerging evidence highlights how socioeconomic position, insurance status, education level, and violence exposure are associated with poor health outcomes for women.^3^ Understanding the factors underlying trends in gynecologic emergency diagnoses requires a more comprehensive examination of SDoH.

Pelvic inflammatory disorder is a broad group of inflammatory conditions of the female genital tract and the surrounding tissues, typically caused by an ascending infection from the endocervix. Approximately 85% of ascending gynecologic infections are due to sexually transmitted infections (STI), most commonly *Neisseria gonorrhoeae* or *Chlamydia trachomatis*.^4^ Infection may be localized to the cervix (cervicitis) or uterus (endometritis), or it can ascend the reproductive tract to the fallopian tubes (salpingitis) or the ovaries (oophoritis). These infections can lead to complications such as the development of tubo-ovarian abscesses or adhesive disease, including partial or total obstruction of the fallopian tubes, which may require surgical intervention. We used the *International Classification of Diseases, 10^th^ Revision* (*ICD-10*) code N70 representing salpingitis and oophoritis for analysis, as patients with identified ascending reproductive infections are higher acuity and often require more intensive treatment through hospitalization or surgery, compared to cervicitis (N72) or endometritis (N71). Salpingitis and oophoritis can also lead to lasting reproductive harm, including chronic pelvic pain, infertility, and increased risk of ectopic pregnancy.^5^,^6^ Using code N70 enabled us to focus on higher acuity, ascending infections that may otherwise have been missed by analysis of the broader spectrum of pelvic inflammatory disorder (N73.9).

Previous research has examined how race influences salpingitis and oophoritis outcomes and has shown that Black women 20–39 years of age have the highest hospitalization rates compared to other racial groups for salpingitis and oophoritis.^7^ Within the same study, Black women had the lowest proportion of hospitalizations associated with hysterectomy, suggesting potential differences in treatment approaches or access to care between racial groups.^7^ Moreover, in addition to Black race, low socioeconomic status and poor income status have also been identified as risk factors for *C trachomatis* infection, a primary cause of salpingitis.^8^,^9^

**Population Health Research Capsule**

What do we already know about this issue?

*Numerous studies have shown the negative impact of social determinants of health (SDoH) on women’s health and their respective clinical trajectory*.

What was the research question?

*Can ICD-10 Z codes be used to study future clinical outcomes following an initial emergency department (ED) encounter for severe pelvic inflammatory disease?*

What was the major finding of the study?

*58% of patients with SDoH Z codes revisited the ED compared to 45% without (P < .001, 95% CI, 1.222–1.355)*.

How does this improve population health?

*With more consistent documentation, ICD-10 Z codes could be used to study discrepancies in clinical care, which can help underserved populations*.

Many patients with salpingitis and oophoritis initially present to the emergency department (ED), particularly those with limited access to regular primary or reproductive healthcare.^10^ Despite this, existing literature primarily focuses on inpatient hospitalizations and is limited in its discussion of how SDoH disparities impact early diagnostic and management decisions among ED patients.^10^ The *ICD-10* Z codes (Z55–Z65), which document adverse SDoH in the electronic health record (EHR), are becoming more accessible and present an opportunity to recognize patients experiencing social risk, particularly within emergency care settings.^11^ As they currently stand, however, these codes are underused and lack widespread incorporation despite the potential benefits of identifying SDoH in preventing adverse healthcare outcomes,^12^ even though they offer a standardized way to document important social risk factors that are otherwise challenging to measure.^12^–^15^ Furthermore, the use of SDoH codes enables the identification of modifiable risk factors and supports the development of interventions to reduce disparities in salpingitis and oophoritis outcomes, particularly among under-represented and high-risk groups.^16^

By leveraging SDoH Z codes, our research aligns with national priorities to improve SDoH data infrastructure and supports the broader movement toward integrating social context into risk stratification and outcome assessment in the female population.^13^,^15^,^17^ Our primary outcome in this study was to evaluate whether the presence or absence of documented SDoH Z codes during an initial ED visit for salpingitis and oophoritis was associated with differences in subsequent healthcare utilization, management, and clinical outcomes following that encounter. We sought to quantify these associations by calculating relative risks (RR).

## METHODS

### Study Design

We used de-identified EHR data from the TriNetX Research Network database in this retrospective, propensity-matched cohort study. TriNetX is a federated health research platform that aggregates clinical data from 106 healthcare organizations; the data available through this platform include standardized records of diagnoses, procedures, medications, laboratory results, and demographics, all encoded using *ICD-10*, Current Procedural Terminology (CPT), and RXNorm coding systems.

### Study Participants

The study population included female patients 18–49 years of age who presented to an ED between January 1, 2000–January 1, 2024, and were subsequently diagnosed with salpingitis and oophoritis (*ICD-10* code N70). This age group was selected based on the WHO definition of reproductive-age females.^1^ We extracted data on May 29, 2025, and constructed two distinct cohorts. Cohort 1 consisted of patients who presented to the ED (CPT code 1013711) and were subsequently diagnosed with salpingitis and oophoritis while having at least one SDoH-related *ICD-10* Z code (Table 1). Cohort 2 consisted of patients who presented to the ED with salpingitis and oophoritis without any SDoH Z code documentation (Table 1).

All patients were required to have had at least one subsequent clinical encounter within five years of the index ED visit to ensure the patient had an active medical record. We defined the index event as the day the patient presented to the ED and was diagnosed with salpingitis and oophoritis. Study outcomes were analyzed over the one-year period following the index event, beginning on the day after the index ED visit. We began assessing outcomes starting the day after the ED visit to allow sufficient time for diagnostic confirmation and initiation of treatment, thereby minimizing misclassification of outcomes that may have occurred during the initial encounter.

### Outcome Measurements

Our primary outcome measure was the relative risk of variables involving subsequent healthcare utilization, management, and clinical outcomes as documented in future encounters, following the index encounter for salpingitis and oophoritis. Outcome variables encompassed clinical, procedural, pharmacologic, and psychosocial domains, with a critical differentiation between outcomes evaluated as incident cases vs those considered regardless of patient medical history. For new outcomes, we excluded from these analyses patients with prior documentation of the diagnosis or procedure before the index ED visit. These outcomes included surgical procedures commonly associated with salpingitis and oophoritis: laparoscopic procedures (CPT 1008895); drainage of ovarian abscess (CPT 1008919); oophorectomy (CPT 1014213); ovarian and fallopian tube excisions (CPT 1008905, *ICD-10*-PCS 0UB0-0UB2, 0UB5-0UB7); resection (*ICD-10*-PCS 0UT0-0UT2, 0UT5-0UT7); and drainage (*ICD-10*-PCS 0U90-0U902, 0U905-0U907); and acquired absence of ovaries (Z90.72).

Additionally, we analyzed as new outcomes any psychiatric and behavioral health diagnoses that emerged post-index event, including depressive episodes (F32), post-traumatic stress disorder (PTSD) (F43.1), anxiety and stress-related disorders (F40–F48), opioid use-related disorders (F11), and nicotine dependence (F17). Additional post-index diagnostic outcomes examined under this criterion were irregular menstruation (N92.6); labeling of the patient as “medically noncompliant” (Z91.1); follow-up for sterilization care (Z30.2); fertility testing (Z31.41); and infertility (N97.0, N97.1, N97.9). Outcomes not limited to new occurrences included prescription of specific medications, such as acetaminophen (RxNorm 161), ibuprofen (RxNorm 5640), ketorolac (RxNorm 35827), opioid analgesics (VA code CN101), other analgesics and antipyretics (VA code N02B), antiemetics (VA code GA605), and glucocorticoids (VA code HS051). Additional outcomes in this category included clinical complications such as sepsis (A41 or A40), postprocedural infections (T81.4), peritonitis (K65), and acute parametritis and pelvic cellulitis (N73.0).

We also captured measures of healthcare without restricting to first-time events. These included hospital admissions (inpatient encounter code “Visit:Inpatient Encounter” or CPT 1013659); return visits to the ED (EMER code “Visit: Emergency Department” or CPT 1013711); critical care services (CPT 1013729); gynecologic follow-up (*ICD-10* Z01.4); follow-up for contraception care (*ICD-10* Z30.01 or Z30.430); and STI screening (Z11.3).

### Statistical Analysis

We performed all analyses using built-in statistical capabilities of the TriNetX platform. For each outcome, risk-based comparisons were made to evaluate the proportion of affected individuals across the two cohorts. We reported findings as risk estimates with corresponding risk differences, risk ratios, and 95% confidence intervals. Where clinically appropriate, patients with a documented history of the outcome prior to the index event were excluded to focus on incident presentations. Each outcome was evaluated over the one-year follow-up period at the following intervals: 1 day – 1 month; 1 month – 6 months; 6 months – 1 year; and 1 day – 1 year. To address potential confounding, we used propensity score matching. Matching was completed in a 1:1 ratio using logistic regression-derived propensity scores based on baseline characteristics including age, race, ethnicity, and comorbidities including hypertensive diseases (I10–I1A), diabetes mellitus (E08–E13), obesity (E65–E68), mental health disorders (F01–F99), and tobacco use (Z72.0).

### Ethical Considerations

The study protocol was reviewed and approved as exempt research by the Penn State Institutional Review Board (STUDY00027222), and all data handling complied with Health Insurance Portability and Accountability Act research standards.

## RESULTS

### Baseline Patient Characteristics

Before matching, Cohort 1 consisted of 2,793 patients and Cohort 2 included 20,633 patients. The proportion of this initial cohort that had at least one SDoH Z code was 11.9%. Table 2 demonstrates how, compared to the non-SDoH cohort, patients in the SDoH group were more frequently identified as Black, American Indian or Alaskan Native, not Hispanic or Latino, and had a higher prevalence of all comorbid conditions included in this analysis (all *P* < .001). Following propensity score matching, we included in the analysis 5,570 patients (50% with documented Z codes and 50% without). Post-matching baseline characteristics demonstrated strong covariate balance with no statistically significant differences in age, race, ethnicity, or comorbidity burden between the groups (all *P*-values > .1), indicating successful matching. All reported relative risk ratios in the subsequent analysis reflect the relative likelihood of outcomes among patients with SDoH Z code documentation, as compared to their matched counterparts without such documentation.

### Outcomes From 1 Day To 1 Month Post-Index Visit

During the first 30 days following the index ED visit, patients with SDoH Z code documentation were significantly less likely to undergo surgical intervention (RR, 0.589; 95% CI, 0.454–0.765, *P* < .001), receive analgesic medications including acetaminophen (RR, 0.915; 95% CI, 0.851–0.983, *P* = .02), opioids (RR, 0.874; 95% CI, 0.814–0.938, *P* < .001), ibuprofen (RR 0.849; 95% CI, 0.768–0.938, *P* = .001), and ketorolac (RR, 0.773, 95% CI, 0.681–0.876, *P* < .001). They were also less likely to be diagnosed with peritonitis (RR, 0.432; 95% CI, 0.232–0.805, *P* = .007) relative to patients without documented SDoH Z codes.

Furthermore, patients with documented SDoH Z codes were significantly more likely to revisit the ED (RR, 1.228; 95% CI, 1.097–1.376, *P* < .001); require hospital admission (RR, 1.252; 95% CI, 1.145–1.368, *P* < .001); receive a new diagnosis of anxiety (RR, 1.873; 95% CI, 1.130–3.103, *P* = 0.01); use critical care services (RR, 1.846; 95% CI, 1.149–2.967, *P* = .01); and undergo STI screening (RR, 2.857; 95% CI, 1.743–4.683, *P* < .001) compared to patients without any recorded SDoH Z codes (Table 3).

### Outcomes From 1 Month To 6 Months Post-Index Visit

Between one and six months following the index ED visit, patients with documented SDoH Z codes exhibited a significantly increased likelihood of returning to the ED (RR, 1.498; 95% CI, 1.379–1.627, *P* < .001); being admitted to the hospital (RR, 1.421; 95% CI, 1.258–1.605, *P* < .001); and developing sepsis (RR, 1.759, 95% CI, 1.118–2.766, *P* = .01). This group was also more frequently prescribed analgesic medications such as acetaminophen (RR, 1.234; 95% CI, 1.124–1.356, *P* < .001), opioids (RR, 1.143; 95% CI, 1.043–1.252, *P* = .004), ketorolac (RR, 1.209; 95% CI, 1.062–1.377, *P* = .004), and other analgesics (RR, 1.293; 95% CI, 1.185–1.412, *P* < .001). Likewise, this group showed a higher rate of being prescribed antiemetics (RR, 1.181; 95% CI, 1.073–1.299, *P* < .001).

Mental and behavioral health diagnoses also occurred at greater incidence among the SDOH group, with higher rates of anxiety (RR, 1.524, 95% CI, 1.070–2.169, *P* = .02); depressive episodes (RR, 1.670, 95% CI, 1.116–2.500, *P* = .01); PTSD (RR, 2.779, 95% CI, 1.332–5.799, *P* = .004); follow-up for sterilization (RR, 2.620, 95% CI, 1.261–5.444, *P* = .007), and medical noncompliance (RR, 2.557, 95% CI, 1.261–5.185, *P* = .007). Additionally, patients with documentation of SDoH Z codes showed higher frequency of STI screening compared to patients without documented SDoH Z codes (RR, 1.816; 95% CI, 1.428–2.311, *P* < .001). In contrast, patients with documented SDoH Z codes were less likely to undergo surgical procedures (RR, 0.619; 95% CI, 0.477–0.805, *P* < .001) in this timeframe compared to patients without documented SDoH Z codes.

### Outcomes From 6 Months To 1 Year Post-Index Visit

From six months to one year post-index visit, patients with documented SDoH Z codes continued to exhibit higher rates of ED returns (RR, 1.488; 95% CI, 1.373–1.614, *P* < .001); hospital admissions (RR, 1.597; 95% CI, 1.396–1.828, *P* < .001); sepsis diagnosis (RR, 2.722; 95% CI, 1.590–4.660, *P* < .001); and use of critical care services (RR, 2.13; 95% CI, 1.30–3.487, *P* = .002) compared to those without SDoH Z code documentation. Analgesic use also remained elevated, including acetaminophen (RR, 1.357, 95% CI, 1.225–1.503, *P* < .001), opioids (RR, 1.286; 95% CI, 1.163–1.422, *P* < .001), ibuprofen (RR, 1.319, 95% CI, 1.140–1.526, *P* < .001), ketorolac (RR, 1.301; 95% CI, 1.125–1.506, *P* < .001), and other analgesics (RR, 1.379; 95% CI, 1.254–1.516, *P* < .001).

This cohort also showed higher likelihood of receiving antiemetic medications (RR, 1.424, 95% CI, 1.282–1.582, *P* < .001), glucocorticoids (RR, 1.424; 95% CI, 1.267–1.600, *P* < .001), and STI screening (RR, 1.541; 95% CI, 1.235–1.924, *P* < .001). Patients with SDoH Z codes also remained at higher risk of experiencing depressive episodes (RR, 2.291; 95% CI, 1.484–3.535, *P* < .001) and PTSD (RR, 2.682, 95% CI, 1.279–5.624, *P* = .007) compared to those without SDoH Z code. No statistically significant increases in any outcomes were observed among patients lacking documented SDoH Z codes during this timeframe.

### Outcomes From 1 Day To 1 Year Post-Index Visit

Across the full one-year period, cumulative outcomes further highlighted disparities between cohorts. Patients with SDoH Z codes demonstrated a significantly higher likelihood of ED revisits (RR, 1.287; 95% CI, 1.222–1.355, *P* < .001); hospital admissions (RR, 1.333; 95% CI, 1.248–1.423, *P* < .001); and use of critical care services (RR, 1.757; 95% CI, 1.317–2.345, *P* < .001) compared to patients without SDoH Z code documentation. They were also more frequently screened for STIs (RR, 1.657; 95% CI, 1.410–1.948, *P* < .001). Regarding medication use, patients with SDOH Z codes were more likely to receive prescriptions for acetaminophen (RR, 1.048, 95% CI, 1.000–1.099, *P* = .05), antiemetics (RR, 1.083, 95% CI, 1.030–1.140, *P* = .002), glucocorticoids (RR, 1.114; 95% CI, 1.041–1.192, *P* = .002), and other analgesics (RR, 1.078; 95% CI, 1.031–1.127, *P* < .001).

Mental health and behavioral outcomes remained elevated within this population, with significantly increased risks of depressive episodes (RR, 1.890; 95% CI, 1.432–2.495, *P* < .001); PTSD (RR, 3.026; 95% CI, 1.897–4.826, *P* < .001); follow-up for sterilization (RR, 3.420; 95% CI, 1.923–6.084, *P* < .001); and medical noncompliance (RR, 1.741; 95% CI, 1.175–2.581, *P* = .005). Likewise, rates of opioid use-related disorders (RR, 2.533; 95% CI, 1.440–4.456, *P* < .001) and anxiety (RR, 1.565; 95% CI, 1.241–1.973, *P* < .001) were significantly higher compared to patients without SDoH Z codes. On the contrary, patients with SDoH Z codes were significantly less likely to undergo surgical procedures (RR, 0.679; 95% CI, 0.577–0.799, *P* < .001); be diagnosed with peritonitis (RR, 0.602; 95% CI, 0.390–0.943, *P* = .03); and have an encounter or fertility testing (RR, 0.416; 95% CI, 0.212–0.812, *P* = .008) during the same period (Table 4).

## DISCUSSION

This study found that among patients presenting to the ED with salpingitis and oophoritis, those with documented SDoH *ICD-10* Z codes exhibited increased healthcare utilization compared to patients without documented SDoH Z codes. Before propensity score matching, the SDoH cohort was more likely to be Black, American Indian or Alaska Native, not Hispanic or Latino, and had a higher prevalence of all comorbid conditions used for propensity score matching in this analysis. Although both groups were matched by demographics and key comorbidities, patients with documented SDoH Z codes experienced increased hospital admissions and ED utilization during the year following their index visit, were less likely to undergo surgical intervention, had unequal analgesic patterns, and showed elevated incidence of new mental and behavioral health diagnoses. Given the known underuse of SDoH *ICD-10* Z codes in clinical practice, the true magnitude of disparities is likely underestimated in this study’s findings.

Our results showing increased repeat ED visits and hospitalizations, as well as increased requirement for critical care services in patients with documented SDoH Z codes, are consistent with prior studies highlighting disparities in healthcare access and usage among socioeconomically disadvantaged groups.^18^,^19^ Patients facing challenges such as a lack of health insurance, unstable housing and transportation, inadequate health literacy, or lower social support have more difficulty maintaining follow-up care, which increases the likelihood of returning to the ED.^18^,^20^,^21^ The increased rates of hospital admissions and critical care services among the SDoH cohort likely indicate that they are more likely to present to the ED with more severe pathology when compared to those without SDoH Z codes, possibly due to disparities related to lack of access to primary care.^18^ This lack of access to primary care services in the outpatient setting may also explain the increased prevalence of STI testing in patients with documented SDoH Z codes in the ED.^22^–^24^ Moreover, our results showed that patients with SDoH Z codes were less likely to have an encounter for fertility testing following their ED visit, which further illustrates discrepancies in access to specialized care and the influence of SDoH on women’s health as a whole.

Another key finding demonstrated that patients with SDoH Z codes were less likely to receive surgery. This may indicate financial or insurance-related concerns, which have previously been shown to limit access to surgical care.^25^,^26^ This may also represent a different threshold for hospital admission or timeliness of seeking medical care. Additionally, a physician’s implicit bias in treatment of individuals with SDoH may lead them to underestimate the severity of disease presentation and progression.^27^–^29^

An important finding of this study was the difference in pain medication administration between patients with and without recorded SDoH Z codes. Patients with SDoH Z code documentation were less frequently prescribed pain medications within the first month after their index visit. Thus, SDoH factors could be a contributing force driving inequitable treatment across patient populations in the ED. This finding is consistent with prior studies describing how emergency physicians minimize pain in individuals from marginalized or high-risk populations, contributing to overall disparities in pain management of gynecologic conditions.^30^,^31^ After one month, patients with recorded SDoH codes became more frequently prescribed analgesics than the non-SDoH cohort. Although difficult to definitively conclude, this may represent inadequate management of gynecologic concerns at the index visit, leading to prolonged discomfort and increased morbidity in the SDoH positive population.

This study also underscores the unequal burden of newly diagnosed mental health conditions among patients with documented SDoH Z codes. Results showed that patients with documented SDoH Z codes were at higher risk of developing anxiety, depressive episodes, and PTSD in the next year following their initial ED visit. This finding aligns with previous evidence describing how individuals experiencing SDoH were associated with higher odds of being diagnosed with major depression and anxiety disorders than their non-SDoH counterparts.^32^,^33^

Overall, this paper shows that research analysis based on documented *ICD-10* Z codes for SDoH factors mirrors existing literature in illustrating the negative relationship between women’s health and SDoH. These *ICD-10* Z codes provide standardized documentation for important social risk factors and should be given additional consideration in physician’s documentation efforts to accurately record the elements that may contribute to worse outcomes. The ED environment is often overburdened due to boarding and a high volume of acute patients. As a result, adequate time may not be taken to properly document relevant SDoH Z codes at discharge. Clinicians understandably prioritize their clinical duties, and less attention may be invested into properly documenting SDoH Z codes compared to diagnosis codes. Many clinicians may lack understanding or knowledge of SDoH Z codes and additionally may lack the training to incorporate these codes into their clinical practice. Unfortunately, clinicians and hospital systems are currently not incentivized to properly document these SDoH Z codes as they minimally impact reimbursement. However, with improved utilization, *ICD-10* Z codes may give physicians additional data points, allowing them to incorporate social context into management of their patients. Further investigation into the utility of *ICD-10* codes as a research tool is warranted, given the broad applicability of their use in statistical analysis to help elucidate relationships which may be driving adverse health outcomes.

## LIMITATIONS

This research drew on a pre-existing dataset, which may contain incomplete or inaccurate entries. One limitation lies in the dependence on *ICD-10* Z codes to identify individuals with documented SDoH. The SDoH codes are inconsistently and infrequently recorded across healthcare systems. This may contribute to misclassification, an under-representation of the true SDoH burden, and potential selection bias in the analysis. In this paper, the absence of documented SDoH codes is assumed to correspond with the lack of patient SDoH factors. However, this absence could be explained by the lack of proper documentation. Moreover, the accuracy and thoroughness of SDoH data are influenced by variations in screening protocols, differences in clinician practices, and institutional discrepancies. These factors can lead to non-random data omissions and limit generalizability.

The less frequent use of SDoH Z codes may represent significant limitations to this study. Within the cohort structure of this study, the control group without documented SDoH Z codes is assumed not to have SDoH factors present. However, there is a significant possibility that SDoH factors were present but not documented among this cohort simply due to a lack of Z code use, which may impact the associations observed. Prior research shows that SDoH codes are used in < 2% of inpatient discharges, despite a much higher estimated prevalence of adverse social conditions among hospitalized patients.^13^^,35^ Ultimately, the low uptake of SDoH Z codes in clinical documentation introduces potential bias into EHR-based studies in general and may limit the generalizability of the results of this paper. Underuse of these diagnostic codes may lead to underestimation of the impact of SDoH in this patient population.

While propensity score matching was used to balance all covariables, unmeasured influences may still confound the observed relationships. These factors may include patient health literacy, individual preferences, or the presence of community support services. Additionally, due to the use of a de-identified dataset compiled from various healthcare institutions, it was not possible to adjust for site-specific variations in SDoH documentation, ED staffing models, or local referral systems.

## CONCLUSION

This study demonstrated that patients presenting to EDs with salpingitis and oophoritis with documented Z codes for social determinants of health experience increased healthcare use compared to patients without these documented codes. Documented SDoH Z codes were associated with increased risk of ED repeat visits, hospitalization, need for critical care, and higher incidence of new mental health conditions. These findings highlight the clinical relevance of SDoH in influencing acute care utilization and patient outcomes, underscoring the importance of routine screening and documentation of SDoH in electronic health records. Addressing underlying social needs may be a key strategy in reducing healthcare burden and improving long-term outcomes for vulnerable populations.

## Figures and Tables

**Table 1:** *International Classification of Diseases, 10^th^ Revision*, Z codes, and their descriptions, used in a study comparing female patients with and without documented social determinants of health who presented to the emergency department with salpingitis and oophoritis.

*ICD-10*	Description
Z55	Problems related to education and literacy
Z56	Problems related to employment and unemployment
Z57	Occupational exposure to risk factors
Z58	Problems related to physical environment
Z59	Problems related to housing and economic circumstances
Z60	Problems related to social environment
Z62	Problems related to upbringing
Z63	Other problems related to primary support group, including family circumstances
Z64	Problems related to certain psychosocial circumstances
Z65	Problems related to other psychosocial circumstances

*ICD-10, International Classification of Diseases, 10^th^ revision*.

**Table 2:** Baseline characteristics before and after propensity score matching in a study comparing female emergency department patients diagnosed with salpingitis and oophoritis with and without documented social determinants of health. Cohort 1: Patients who had a documentation of both salpingitis and oophoritis and one or more SDoH *ICD-10* codes. Cohort 2: Patients with salpingitis and oophoritis and no documentation of an SDoH *ICD-10* code.

Before propensity score matching			After propensity score matching				
	
Patients (n)	Age at Index	*P*-value	Patients (n)		Age at Index		*P*-value
	Mean (SD)				Mean (SD)		

2,793	32.7 (7.8)	< .001	2,785		32.7 (7.8)		.90
20,633	32.0 (7.7)		2,785		32.7 (7.8)		

Characteristic		Before propensity score matching			After propensity score matching		
	
		Patients (n)	% of Cohort	*P*-value	Patients (n)	% of Cohort	*P*-value
1	White	1,270	45.5%	< .001	1,269	45.6%	.85
2		10,224	49.8%		1,262	45.3%	
1	American Indian or Alaska Native	28	1.0%	< .001	27	1.0%	.47
2		71	0.3%		22	0.8%	
1	Unknown race	143	5.1%	.002	143	5.1%	.86
2		1,369	6.7%		140	5.0%	
1	Native Hawaiian or other Pacific Islander	49	1.8%	.77	49	1.8%	.92
2		345	1.7%		50	1.8%	
1	Unknown ethnicity	449	16.1%	< .001	447	16.1%	.66
2		4,083	19.9%		435	15.6%	
1	Not Hispanic or Latino	1,979	70.9%	< .001	1,976	71.0%	.72
2		13,700	66.7%		1,988	71.4%	
1	Hispanic or Latino	362	13.0%	.50	362	13.0%	1
2		2,762	13.4%		362	13.0%	
1	Black	1,150	41.2%	< .001	1,147	41.2%	.63
2		6,913	33.6%		1,165	41.8%	
1	Other race	101	3.6%	.24	101	3.6%	.94
2		840	4.1%		100	3.6%	
1	Asian	49	1.8%	< .001	49	1.8%	.76
2		783	3.8%		46	1.7%	
1	I10-I1A: Hypertensive diseases	132	4.7%	< .001	128	4.6%	.36
2		453	2.2%		114	4.1%	
1	E08-E13: Diabetes mellitus	85	3.0%	< .001	82	2.9%	.18
2		247	1.2%		66	2.4%	
1	E65-E68: Overweight, obesity, and other hyperalimentation	121	4.3%	< .001	119	4.3%	.54
2		503	2.4%		110	3.9%	
1	F01-F99: Mental, behavioral, and neurodevelopmental disorders	412	14.8%	< .001	407	14.6%	.82
2		1,221	5.9%		413	14.8%	
1	Z72.0: Tobacco use	19	0.7%	< .001	17	0.6%	.86
2		57	0.3%		16	0.6%	

*ICD-10*, *International Classification of Diseases, 10^th^ Revision*; *SDoH*, social determinants of health.

**Table 3:** Outcomes from 1 day to 1 month post-index visit in a study comparing female emergency department patients with salpingitis and oophoritis with and without documented social determinants of health.

Outcome	+ SDOH n (%)	− SDOH n (%)	*P*-value	Risk ratio (95% CI)
Surgery	84 (4.0%)	147 (6.9%)	< .001	0.589 (0.454, 0.765)
ED return	549 (19.7%)	447 (16.1%)	< .001	1.228 (1.097, 1.376)
Hospital admission	806 (28.9%)	644 (23.1%)	< .001	1.252 (1.145, 1.368)
Complication following procedure	67 (2.4%)	72 (2.6%)	.67	0.931 (0.670, 1.292)
Acetaminophen	926 (33.3%)	1,012 (36.3%)	.02	0.915 (0.851, 0.983)
Opioids	924 (33.2%)	1,057 (38.0%)	< .001	0.874 (0.814, 0.938)
Ibuprofen	555 (19.9%)	654 (23.5%)	.001	0.849 (0.768, 0.938)
Ketorolac	367 (13.2%)	475 (17.1%)	< .001	0.773 (0.681, 0.876)
Sepsis	67 (2.4%)	86 (3.1%)	.12	0.779 (0.569, 1.068)
Infection following procedure	26 (0.9%)	33 (1.2%)	.36	0.788 (0.473,1.314)
Opioid related disorders	12 (0.5%)	10 (0.4%)	.55	1.292 (0.559, 2.985)
Nicotine dependence	12 (0.8%)	21 (1.0%)	.44	0.757 (0.373, 1.533)
Depressive episode	19 (1.4%)	21 (1.0%)	.24	1.441 (0.778, 2.670)
Anxiety	30 (2.8%)	29 (1.5%)	.01	1.873 (1.130, 3.103)
PTSD	16 (0.7%)	10 (0.4%)	.12	1.844 (0.839, 4.056)
GYN follow-up	52 (1.9%)	42 (1.5%)	.30	1.238 (0.827, 1.853)
Critical care services	48 (1.7%)	26 (0.9%)	.01	1.846 (1.149, 2.967)
Other analgesics	991 (35.6%)	1,044 (37.5%)	.14	0.949 (0.886, 1.017)
Antiemetics	807 (29.0%)	807 (29.0%)	1.00	1.000 (0.921, 1.086)
Peritonitis	14 (0.5%)	33 (1.2%)	.007	0.432 (0.232, 0.805)
F/u for contraception	24 (0.9%)	23 (0.8%)	.88	1.043 (0.590, 1.844)
F/u for sterilization	10 (0.4%)	10 (0.4%)	.92	1.047 (0.437, 2.511)
Noncompliance	13 (0.5%)	10 (0.4%)	.36	1.461 (0.642,3.325)
STI screening	60 (2.2%)	21 (0.8%)	< .001	2.857 (1.743, 4.683)
Parametritis and pelvic cellulitis	29 (1.2%)	29 (1.1%)	.73	1.094 (0.656, 1.824)
Glucocorticoids	408 (14.7%)	379 (13.6%)	.27	1.077 (0.946, 1.225)
Encounter for fertility testing	10 (0.4%)	16 (0.6%)	.26	0.636 (0.289, 1.398)
Infertility diagnosis	10 (0.4%)	10 (0.4%)	.99	1.004 (0.419, 2.409)
Irregular menstruation	10 (0.5%)	13 (0.6%)	.81	0.906 (0.398, 2.061)

*GYN*, gynecologic; *PTSD*, post-traumatic stress disorder.

**Table 4:** Outcomes from one day to one year post-index visit in a study comparing emergency department patients with salpingitis and oophoritis with and without documented social departments of health.

Outcome	+ SDOH n (%)	− SDOH n (%)	*P*-value	Risk ratio (95% CI)
Surgery	212 (10.2%)	322 (15.0%)	< .001	0.679 (0.577, 0.799)
ED return	1,619 (58.1%)	1,258 (45.2%)	< .001	1.287 (1.222, 1.355)
Hospital admission	1,274 (45.7%)	956 (34.3%)	< .001	1.333 (1.248, 1.423)
Complication following procedure	125 (4.5%)	134 (4.8%)	.57	0.933 (0.735, 1.183)
Acetaminophen	1,581 (56.8%)	1,508 (54.2%)	.05	1.048 (1.000, 1.099)
Opioids	1,556 (55.9%)	1,569 (56.3%)	.73	0.992 (0.947, 1.039)
Ibuprofen	1,067 (38.3%)	1,089 (39.1%)	.55	0.980 (0.917, 1.047)
Ketorolac	919 (33.0%)	903 (32.4%)	.65	1.018 (0.944, 1.097)
Sepsis	144 (5.2%)	118 (4.2%)	.10	1.220 (0.962, 1.548)
Infection following procedure	49 (1.8%)	55 (2.0%)	.55	0.891 (0.608, 1.305)
Opioid use-related disorders	40 (1.6%)	17 (0.6%)	< .001	2.533 (1.440, 4.456)
Nicotine dependence	61 (4.0%)	82 (4.0%)	.93	0.985 (0.712, 1.363)
Depressive episode	114 (8.4%)	100 (4.6%)	< .001	1.890 (1.432, 2.495)
Anxiety	121 (11.1%)	140 (7.1%)	< .001	1.565 (1.241, 1.973)
PTSD	63 (2.7%)	24 (0.9%)	< .001	3.026 (1.897, 4.826)
GYN follow up	300 (10.8%)	278 (10.0%)	.33	1.079 (0.925, 1.259)
Critical care services	123 (4.4%)	70 (2.5%)	< .001	1.757 (1.317, 2.345)
Other analgesics	1,682 (60.4%)	1,560 (56.0%)	< .001	1.078 (1.031, 1.127)
Antiemetics	1,493 (53.6%)	1,378 (49.5%)	.002	1.083 (1.030, 1.140)
Peritonitis	31 (1.2%)	52 (2.0%)	.03	0.602 (0.390, 0.943)
Follow-up for contraception	117 (4.2%)	96 (3.4%)	.14	1.219 (0.935, 1.588)
Follow-up for sterilization	49 (1.9%)	15 (0.6%)	< .001	3.420 (1.923, 6.084)
Noncompliance	62 (2.6%)	40 (1.5%)	.005	1.741 (1.175, 2.581)
STI screening	353 (12.7%)	213 (7.6%)	< .001	1.657 (1.410, 1.948)
Parametritis and pelvic cellulitis	60 (2.6%)	58 (2.3%)	.50	1.131 (0.792, 1.616)
Glucocorticoids	1,103 (39.6%)	990 (35.5%)	.002	1.114 (1.041, 1.192)
Encounter for fertility testing	12 (0.5%)	29 (1.1%)	.008	0.416 (0.212, 0.812)
Infertility diagnosis	41 (1.7%)	53 (2.2%)	.24	0.787 (0.526, 1.170)
Irregular menstruation	65 (3.3%)	85 (3.7%)	.52	0.900 (0.656, 1.237)

*GYN*, gynecologic; *PTSD*, post-traumatic stress disorder; *SDOH*, social determinants of health; *STI*, sexually transmitted infection.
